# Temozolomide followed by combined immunotherapy with GM-CSF, low-dose IL2 and IFN*α* in patients with metastatic melanoma

**DOI:** 10.1038/sj.bjc.6600717

**Published:** 2003-01-28

**Authors:** G C de Gast, D Batchelor, M J Kersten, F A Vyth-Dreese, J Sein, W F van de Kasteele, W J Nooijen, O E Nieweg, M A de Waal, W Boogerd

**Affiliations:** 1 Division of Med. Oncology, Netherlands Cancer Institute, Amsterdam, The Netherlands; 2 Division of Immunology, Netherlands Cancer Institute, Amsterdam, The Netherlands; 3 Division of Clinical Chemistry, Netherlands Cancer Institute, Amsterdam, The Netherlands; 4 Division of Surgical Oncology, Netherlands Cancer Institute, Amsterdam, The Netherlands; 5 Division of Biometrics, Netherlands Cancer Institute, Amsterdam, The Netherlands

**Keywords:** temozolomide, immunotherapy, malignant melanoma

## Abstract

The purpose of this study is to determine the toxicity and efficacy of temozolomide (TMZ) p.o. followed by subcutaneous (s.c.) low-dose interleukin-2 (IL2), granulocyte–monocyte colony stimulating factor (GM-CSF) and interferon-*α* 2b (IFN*α*) in patients with metastatic melanoma. A total of 74 evaluable patients received, in four separate cohorts, escalating doses of TMZ (150–250 mg m^−2^) for 5 days followed by s.c. IL2 (4 MIU m^−2^), GM-CSF (2.5 *μ*g kg^−1^) and IFN*α* (5 MIU flat) for 12 days. A second identical treatment was scheduled on day 22 and cycles were repeated in stable or responding patients following evaluation. Data were analysed after a median follow-up of 20 months (12–30 months). The overall objective response rate was 31% (23 out of 74; confidence limits 20.8–42.9%) with 5% CR. Responses occurred in all disease sites including the central nervous system (CNS). Of the 36 patients with responding or stable disease, none developed CNS metastasis as the first or concurrent site of progressive disease. Median survival was 252 days (8.3 months), 1 year survival 41%. Thrombocytopenia was the primary toxicity of TMZ and was dose- and patient-dependent. Lymphocytopenia (grade 3–4 CTC) occurred in 48.5% (34 out of 70) fully monitored patients following TMZ and was present after immunotherapy in two patients. The main toxicity of combined immunotherapy was the flu-like syndrome (grade 3) and transient liver function disturbances (grade 2 in 20, grade 3 in 15 patients). TMZ p.o. followed by s.c. combined immunotherapy demonstrates efficacy in patients with stage IV melanoma and is associated with toxicity that is manageable on an outpatient basis.

Metastatic melanoma is a highly chemotherapy-resistant tumour. The best-known single agent dacarbazine (DTIC) achieves a 15–20% response rate with a median survival of 6–7 months. The addition of other cytotoxic agents does not improve survival (Lakhani *et al*, 1990; Chapman *et al*, 1999). Therefore, interest has shifted to immunotherapy as malignant melanoma is considered to be an immunogenic tumour. Although immunotherapy with high-dose IL2 can induce durable complete remissions in a small proportion of patients (Legha *et al*, 1997), the toxicity requires treatment on an in-patient basis with transfer to the intensive care if toxicity is severe.

Recently, a combination of chemo- and immunotherapy has been investigated in several phase II studies (Khayat *et al*, 1993; Legha *et al*, 1998; O'Day *et al*, 1999; Richards *et al*, 1999) with responses of 40–60%. However, in a recent phase III trial, survival did not improve with the addition of high-dose IL2 and IFN*α* to chemotherapy compared to chemotherapy alone (Rosenberg *et al*, 1999).

The occurrence of brain metastasis is a major clinical issue. Its incidence is up to 75% in patients who die from metastatic melanoma (Amer *et al*, 1978). Brain metastases occur as the first site of progression in a significant proportion of patients with a CR, and in a large majority of patients with a PR following systemic treatment with DTIC or cisplatin as neither of these agents cross the blood–brain barrier (Legha *et al*, 1998; O'Day *et al*, 1999).

Temozolomide (TMZ), a prodrug of the alkylating agent 5-(3 methyltriazen-1-yl) imidazole-4-carboximide (MTIC), is a novel alkylating agent that is similar to DTIC. However, unlike DTIC, it is converted to MTIC under physiologic conditions, and oral administration is possible (Amer *et al*, 1978; Stevens *et al*, 1987).

TMZ has comparable activity to DTIC in melanoma (Middleton *et al*, 2000). In addition, it penetrates the blood–brain barrier, which can be beneficial in preventing or treating CNS metastasis.

One of the concerns with TMZ therapy is the occurrence of lymphocytopenia, which could have detrimental effects in an immunogenic tumour such as melanoma. Therefore, we evaluated the toxicity and efficacy of escalating doses of oral TMZ followed by subcutaneous injections of low-dose IL2, GM-CSF and IFN*α* in a phase I/II trial in patients with metastatic melanoma.

The feasibility and dosing of this combination of immunotherapeutic agents was first tested in a phase I trial (De Gast *et al*, 2000). The combination of these three cytokines was based on the theory of improving antigen presentation with GM-CSF, expanding and activating T cells (LD-IL2+GM-CSF), increasing effector cell function (IFN*α*) and immunosensitising tumour cells by increased expression of HLA and adhesion molecules (IFN*α*). This treatment can be given on an outpatient basis, which is an important factor for patients, especially those with a poor prognosis.

## PATIENTS AND METHODS

### Patients

The study was approved by the Protocol Review Committee of the Netherlands Cancer Institute, which dealt with the ethical aspects. All patients entered on study had metastatic melanoma confirmed by biopsy or cytology and signed informed consent before registration. Eligibility criteria were patients older than 18 years who were able to give informed consent, WHO performance scores of 0,1 or 2, measurable disease, adequate bone marrow function (leucocytes ⩾4.0 nl^−1^, platelets >100 nl^−1^), renal function (creatinine <180 *μ*mol l^−1^) and liver function (transaminases, alkaline phosphatase <3×ULN, in case of liver metastases <5×ULN, bilirubin within normal limits). Patients with brain metastases <2 cm could be included if there was no risk of serious complications.

Exclusion criteria included serious cardiac, pulmonary or metabolic disease, pregnancy or lactation, concurrent systemic immunosuppressive treatment, previous or present autoimmune disease, a-HIV antibodies, symptomatic cerebral oedema and any other uncontrolled cancer. No previous chemotherapy or immunotherapy except adjuvant IFN*α* was allowed.

Characteristics of the 74 evaluable patients are listed in Table 1

**Table 1:** Patient characteristics (*n*=74)

Age (years)	
Median	45
Range	21–70
	
WHO performance status	
0	57
1	13
2	4
	
Male : female ratio	44 : 30
	
Sites of disease	
CNS	13
Liver	25
Bone	21
CNS/liver/bone	40 (54%)
	
M1a	15
M1b	9
M1c	50
	
No. of metastatic sites	
One	20
Two	26
More than two	28
	
Primary tumour	
Skin	66
Ocular	4
Mucosa	4

M1a=patients with distant skin, subcutaneous or lymph node metastases; M1b=patients with lung metastases; M1c=patients with other visceral sites of metastasis or increased LDH (AJCC staging system); CNS=central nervous system.

.

### Treatment

#### Chemotherapy

TMZ (Temodal, provided by Schering-Plough, Maarssen, The Netherlands) was given orally for 5 days at a dose of 150, 200 and 250 mg m^−2^ in cohorts of 6, 18 and 18 patients, respectively. Following evaluation of the dose levels, the following cohort of 32 patients received 200 mg m^−2^ in the first cycle with dose adjustment in subsequent cycles depending on the degree of myelosuppression.

#### Immunotherapy

Low-dose IL2 (Aldesleukin, Proleukin^R^ 4 MIU m^−2^, provided by Chiron BV, Amsterdam, The Netherlands), granulocyte–monocyte colony stimulating factor (GM-CSF) (Molgramostim, Leukomax^R^ 2.5 *μ*g kg^−1^, Schering-Plough, Maarssen, (The Netherlands) and interferon-*α*-2b IFN*α* (Intron A^R^ 5 MIU, Schering-Plough, Maarssen, The Netherlands)) were given as subcutaneous injections for 12 days in the abdominal wall, left leg and right leg, respectively. The right or left arm was used if lymphadenectomy had been performed in one or both lower limbs.

The first cycle of combined immunotherapy was initiated on an in-patient basis with the MTD found in the phase I study (De Gast *et al*, 2000). Patients were observed for toxicity and discharged after 2 or 3 days when the drug dosage was deemed safe for home (outpatient) administration. If grade 4 fever with hypotension or any other unacceptable toxicity was seen, the dose of IL2 was reduced by 50%. Subsequent cycles were all given on an outpatient basis.

#### Dose modification

In the third cohort (250 mg m^−2^) the dose of TMZ was reduced to 200 mg m^−2^ for grade 3 myelosuppression or to 150 mg m^−2^ for grade 4 myelosuppression. TMZ was increased in the fourth cohort from 200 to 250 mg m^−2^ in the second or subsequent cycles if no myelosuppression occurred, or to 225 mg m^−2^ if CTC grade 1 myelosuppression occurred. The dose was maintained if grade 2 myelosuppression occurred and reduced to 150 mg m^−2^ if grade 3 or 4 reversible myelosuppression was present.

The IL2 dose was reduced from 4 to 2 MIU m^−2^ if grade 4 fever with hypotension occurred or when persistent severe malaise, diuretic insensitive weight gain >5% or grade 3 liver dysfunction occurred. GM-CSF was stopped if leukocytes increased above 40 nl^−1^.

#### Supportive measures

Patients routinely received granisetron 1 mg orally 1 h prior to TMZ p.o. to prevent nausea and vomiting. Magnesium oxide 500 mg b.i.d./t.i.d. was given routinely to treat granisetron-induced constipation.

Prior to the first administration of immunotherapy, patients received 0.5 l of NaCl 0.9% intravenously. Paracetamol 1 g p.o. was administered 1 h following the injections, 1 g at the onset of chills and 1 g 4 h later and subsequently when necessary up to 4000 mg day^−1^.

Metoclopramide tablets 10 or 20 mg suppositories were used to treat or prevent nausea during the immunotherapy phase. No systemic steroids were permitted except in emergency situations.

#### Evaluation

Prior to treatment, physical examination (PE), intravenous contrast-enhanced computer tomography (CT) scans of chest, abdomen and pelvis, magnetic resonance imaging (MRI) of the brain and radioactive technetium scan of the bone were performed to determine the extent of the disease. In addition, an electrocardiogram and blood tests for haematology, liver and renal function and an HIV antibody test were performed.

Complete blood counts, differential WBC count, platelet count, serum S100, liver and renal function tests were done at the start of treatment, following 5 days of TMZ, and were repeated after weeks 1 and 3 of the immunotherapy during each cycle. In addition, the absolute number of T cells (CD3^+^, CD4^+^, CD8^+^), NK cells (CD3-CD16+56+), monocytes (CD14+), B cells (CD19+) and activated cells (double staining with HLA-DR) were determined with monoclonal antibodies using the FACS scan flow cytometer as described previously. Soluble IL2-receptor (sIL2R, Eurogenetics, Tessenderlo, Belgium) and sCD8 (T Cell Diagnostics, Cambridge MA, USA) were assayed by ELISA as described (De Gast *et al*, 2000).

#### Response evaluation

Physical examination, CT scans and MRI of the brain were repeated after every 2 cycles in week 7. Metastatic disease was quantified as the sum of products of the perpendicular diameters of marker lesions. All measurable lesions on CT scans were used as marker lesions, and up to 10 subcutaneous nodules. Responses were defined as follows: complete response (CR): disappearance of all measurable disease; partial response (PR): 50% or more reduction in measurable disease with no new lesions; stable disease (SD): less than 25% change in measurable disease and no new lesions; progressive disease (PD): 25% or more increase in measurable disease or the appearance of new lesions, or lost to follow-up owing to deteriorating clinical condition. Responses must have been confirmed by post-treatment evaluation 6 weeks later.

In patients with PD, treatment was stopped and palliative radiotherapy considered. In patients with SD another two cycles were given.

If tumour regression occurred, cycles were continued with a response evaluation every two cycles. If a CR was observed, two subsequent consolidation cycles were given.

#### Statistics

Survival curves were constructed using the Kaplan–Meier technique using the initiation of chemotherapy as the starting point. Levels of circulating cells in peripheral blood and of cytokines measured at various time points and in various groups were compared using Student's *t*-test. *P*-values <0.05 were considered significant.

## RESULTS

### Tumour response

A total of 77 patients were entered into the trial in a single institution (Netherlands Cancer Institute, Amsterdam). Three patients were subsequently excluded. One patient with lymph node enlargement appeared to have Hodgkin's disease and the primary tumour was a dysplastic nevus. Two patients did not start with immunotherapy (one because of rapid progressive disease and the other because of large brain metastases). A total of 74 patients received at least 1 week of immunotherapy following chemotherapy and were evaluable for response. Two patients stopped immunotherapy after 7 days because of rapidly progressive disease or severe malaise. Another eight patients stopped treatment after one full cycle of chemoimmunotherapy because of progressive disease (*n*=7) or severe malaise (*n*=1). None of the patients received prior chemotherapy and seven had received adjuvant IFN*α*.

Responses in the 74 evaluable patients are shown in Table 2

**Table 2:** Response evaluation

**Response**	**No. of patients**	**Median PFS (months)**	**Median OS (months)**
CR	4	4, 9, 30+, 30+	13, 15, 32+, 32+
PR	19 1 sCR (iliacal LN)	5	12 (range 4.5–30+)
SD	13 2 sCR (2×lobectomy)	5	12 (range 7–34+)
PD	38	–	4.5 (range 1–11)
			
Total	74		

sCR=surgical CR; LN=lymph node.

. The overall objective response rate was 31% (23 out of 74, confidence limits 20.8–42.9%). CR was seen in four patients following either the second or fourth cycle of therapy. One patient with a PR and an isolated iliac lymph node (LN) underwent resection of the LN resulting in a surgical CR. Two patients with isolated lung metastasis, who remained stable after four cycles of chemoimmunotherapy, underwent (bi)lobectomy.

Two patients with initial CRs remained tumour free (30+ months) as did one patient after lobectomy (24+ months). In the other patient with lobectomy, an isolated LN metastasis was removed 6 months later and two isolated pancreatic metastases 12 months later (0S 34+ months).

**Figure 1:**
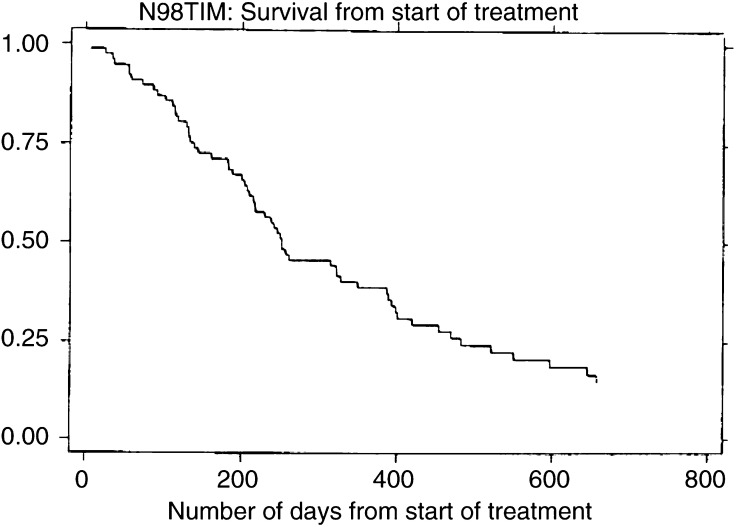
Survival from start of treatment of 74 evaluable patients. Median survival is 252 days.

Survival from the start of treatment is shown in Figure 1

. Median survival is 252 days and the 1 year survival is 41%.

### Central nervous system (CNS) metastases

CNS metastases were present prior to treatment in 13 patients. One patient with a CR in the brain had a lesion of 7.5 mm that disappeared completely. When the mediastinal mass (PR) began to grow again, the brain metastasis remained in remission.

One patient with neoplastic meningitis, who presented with a confusional state, headache, cranial nerve dysfunction and a marked increase of malignant cells in the cerebrospinal fluid, had relief of all symptoms and signs following therapy. Relapse of this meningitis occurred as the first sign of progression, 7 months after the initiation of treatment. Two patients had SD both in and outside of the CNS.

To date, none of the 36 patients with CR, PR or SD have developed CNS metastasis as the first or concurrent site of progression.

### Toxicity TMZ

None of the six patients with 150 mg m^−2^ TMZ×5 or of the first series of 18 patients with 200 mg m^−2^ TMZ developed CTC grade 3 or 4 thrombocytopenia. However, nine out of 18 patients (50%) receiving 250 mg m^−2^ TMZ×5 developed grade 3–4 thrombocytopenia as shown in Table 3

**Table 3:** Grade 3–4 CTC toxicity according to cohorts/dose levels

	**150 mg m^−2^ a**	**200 mg m^−2^ a**	**250 mg m^−2^ a**	**200 mg m^−2^ adj.^a^,^b^**
No. of patients	6	18	18	32
				
Thrombocytopenia				
Grade 3	0	0	5	1
Grade 4	0	0	4	2
				
Lymphocytopenia				
Grade 3	2	5	8	12
Grade 4	0	2	3	3

a Oral administration for 5 days

b Dose adjustments took place in the following cycles according to the degree of myelosuppression.

. Characteristically, thrombocytopenia began on day 16 (d12–d18) and resolved on day 29 (d24–d32). Bleeding did not occur. Platelet transfusions were given to five patients. Based on these results, patients in the fourth cohort received 200 mg m^−2^ TMZ in the first cycle and when appropriate had a dose adjustment in subsequent cycles. Three of 32 patients (9%) developed grade 3 or 4 thrombocytopenia at this dose. Patients with grade 3 or 4 thrombocytopenia generally also developed grade 2 or 3 granulocytopenia. Of the 50 patients who received TMZ 200 mg m^−2^ for 5 days in the first cycle, three (6%) developed grade 3 or 4 thrombocytopenia. A dose reduction of TMZ in subsequent cycles always prevented grade 3 or 4 thrombocytopenia.

Lymphocytopenia grade 3 or 4 occurred in 50% of the patients receiving ⩾200 mg m^−2^ TMZ×5 without a clear effect of the TMZ dose. Severe lymphocytopenia (grade 3 or 4) was present in 13% (nine out of 70) of patients prior to treatment, increased to 49% (34 out of 70) after 5 days of TMZ and was nearly absent after immunotherapy (3%, two out of 70) (Table 4

**Table 4:** Lymphocytopenia before and after treatment of 70 patients fully monitored

	**Before start**	**After 5 days TMZ^a^**	**After immunotherapy**
Grade 0 (>2.0 nl^−1^)	17	3	45
Grade 1 (1.51–2.0)	20	8	15
Grade 2 (1.01–1.5)	24	25	8
Grade 3 (0.51–1.0)	9	27	2
Grade 4 (⩽0.5)	0	7	0

a After 5 days oral TMZ.

).

Lymphocytopenia prior to treatment was not correlated with response, nor did the number of lymphocytes following immuno therapy (results not shown).

### Toxicity immunotherapy

All patients developed flu-like syndrome, fatigue and anorexia. A majority also had transient liver function disturbances, mostly transaminase or alkaline phosphatase elevations (23 grade 1, 20 grade 2, 15 grade 3, 0 grade 4). In 39 out of 74 (52.9%) patients, the IL2 dose was decreased to 2 MIU m^−2^ because of grade IV fever with (rapidly reversible) hypotension (*n*=17), severe fatigue (*n*=7) or grade 3 liver function disturbances (*n*=15). None of the patients died of treatment-related toxicity and none were treated with steroids.

### Immunologic evaluation

**Figure 2:**
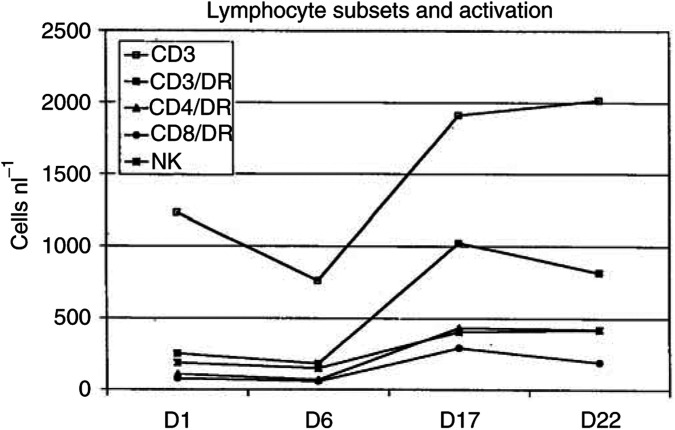
Lymphocyte subsets and activation before start (day 1), following 5 days of TMZ (day 6), following 12 days of immunotherapy (day 17) and prior to start of the second cycle (day 22). T cell (CD3) number decreased by TMZ, but was corrected following immunotherapy. Activated T cells (CD3/DR), its subsets (CD4/DR and CD8/DR) as well as the number of NK cells increased significantly following immunotherapy.

Although depression of CD3^+^ T cells did occur following 5 days of TMZ, it did not prevent activation of CD3, CD4 or CD8+ T cells, nor the expansion of NK cells by immunotherapy (Figure 2

). B-cell numbers were not influenced (results not shown). There was no relation between lymphocyte number and activation prior to or following immunotherapy and response to treatment (data not shown).

Similar to the activated T-cell numbers, soluble IL-2R and soluble CD8 showed no decrease following 5 days TMZ treatment (sIL2R 631 and 835 U ml^−1^, respectively, before and after TMZ, and sCD8 288 and 342 U ml^−1^). A significant increase was seen after 12 days of immunotherapy (sIL2R increased to 9066 U ml^−1^; sCD8 increased to 860 U ml^−1^). No correlation was found with response or survival (data not shown).

## DISCUSSION

Thrombocytopenia was the dose-limiting toxicity (DLT) of TMZ and combined subcutaneous immunotherapy with low-dose IL2, GM-CSF and IFN*α*, and it was dose- and patient-dependent. The highest dose (250 mg m^−2^×5) caused grade 3–4 thrombocytopenia in nine out of 18 patients (50%) *vs* three out of 50 patients (6%) starting with 200 mg m^−2^. Several phase I trials of TMZ in patients with advanced cancer also demonstrated myelosuppression, especially thrombocytopenia, as the DLT (Dhodapkar *et al*, 1997; Hammond *et al*, 1999; Newlands *et al*, 1992; O'Reilly *et al*, 1993). The thrombocytopenia was always transient and bleeding was not observed. Platelet transfusions were given to five patients. A dose reduction of TMZ prevented severe thrombocytopenia in all patients in subsequent cycles.

An interesting, previously not well-documented phenomenon was severe (grade 3 or 4) lymphocytopenia, present in 13% (nine out of 70) of patients before initiation of TMZ, which increased to 49% (34 out of 70) following 5 days of TMZ. To correct the expected lymphocytopenia by TMZ in such an immunogenic tumour as malignant melanoma, we administrated combined immunotherapy with GM-CSF, low-dose IL2 and IFN*α* s.c. for 12 days in a dosing schedule determined in a phase I study (De Gast *et al*, 2000). The combination of GM-CSF and low-dose IL2 was given in order to activate and expand antigen-presenting cells and T cells. Moreover, we aimed to avoid the toxic effects of the activation and expansion of NK cells by high-dose IL2. As NK cells have an IL2-receptor (IL2R) of intermediate affinity, we expected no NK cell activation with this low dose of IL2 in contrast to T cells, which have an IL2R of high affinity when activated (Caligiuri *et al*, 1990; Nagler *et al*, 1990). Following immunotherapy, severe lymphocytopenia was only present in 3% (two out of 70) of patients. Whether this is only an effect of the immunotherapy or reflects also a spontaneous recovery following TMZ is not clear. In addition to lymphocyte recovery, lymphocyte activation was not inhibited when TMZ was followed by immunotherapy as indicated by the increase of HLA-DR-expressing T cells and increased levels of sIL2R and sCD8. A moderate increase in the number of NK cells was observed with the low-dose IL2 combined with GM-CSF.

The toxicity of combined immunotherapy consisted of flu-like syndrome, fatigue, anorexia and transient liver function disturbances. Since the expansion of NK cell numbers as seen in the phase I study was clearly dependent on the IL2 dose (De Gast *et al*, 2000), we decreased the IL2 dose from 4 to 2 MIU m^−2^ when toxicity was severe (grade 3 or 4 CTC), assuming that it was mainly an IL2 effect. CD4-T-cell activation as reflected in sIL2R levels was maintained with this dose (De Gast *et al*, 2000). The IL2 dose was reduced to 2 MIU m^−2^ in 39 out of 74 patients. Owing to the supportive care programme, treatment could be continued on an outpatient basis in all patients, which is important for this patient population with a poor prognosis and short survival.

TMZ has shown efficacy in primary brain tumours and metastatic melanoma in phase II trials (Bleehen *et al*, 1995; Yung *et al*, 1999) However, in a phase III trial, it was not found to be superior to DTIC (Middleton *et al*, 2000). In our study, an overall response rate of 31% (24 out of 74 patients) was achieved with a 1-year survival of 41% and a median survival of 252 days. The response rate was higher than that shown for TMZ alone previously (Middleton *et al*, 2000). Whether the higher response is because of dose intensification of TMZ (cycles 3 weeks^−1^) or the addition of immunotherapy is unclear. Patient selection is less likely to be a factor, as our patient population had an extremely poor prognosis with >50% of patients having CNS, liver or bone metastases, well-known indicators of poor prognosis (Ryan *et al*, 1993). Whether subcutaneous combined immunotherapy adds a survival benefit to monotherapy with TMZ is presently the goal of a multicentre phase III trial, which is now being conducted in the Netherlands. In this respect, it may be important that neither the number of lymphocytes, (activated) T cells or NK cells prior to start nor those following immunotherapy were correlated with response. Recently it has been found that lymphocyte numbers after high dose IL-2 correlated with response (Phan *et al*, 2001). We did not find such a correlation in our study.

A major finding of the study was the effect of TMZ on CNS metastases: in patients with CNS metastases present on MRI, one out of 13 patients showed a complete disappearance of a brain metastasis and continued remission when progression occurred at extracranial sites and one patient demonstrated long-term regression of meningeal metastases. In previous studies of chemo-immunotherapy in metastatic melanoma, no response was observed in brain metastases despite objective tumour regression at extracranial sites (Legha *et al*, 1998; O'Day *et al*, 1999).

Of interest, TMZ might be effective in the prevention of brain metastases, which is one of the most threatening complications of advanced melanoma. In this study, brain metastasis did not develop as the first or concurrent site of progressive disease in patients with either responsive disease (*n*=23) or with stable disease (*n*=13). Two prior published reports have indicated brain metastasis as a first site of progressive disease in four out of nine patients with a complete remission (Legha *et al*, 1998; O'Day *et al*, 1999). Moreover, in a retrospective case-controlled study of responders to DTIC or TMZ, it was found that responders to TMZ developed significantly less CNS relapses than responders to DTIC (Paul *et al*, 2002). Importantly, low-dose IL-2 did not appear to cause signs or symptoms of brain oedema in patients with CNS disease.

In conclusion, temozolomide p.o. followed by combined immunotherapy s.c. demonstrates efficacy in patients with stage IV melanoma and is associated with toxicity manageable on an outpatient basis.
